# Supplementary material for: Genome-Wide Identification of Members of the Soybean CBL Gene Family and Characterization of the Functional Role of GmCBL1 in Responses to Saline and Alkaline Stress
Source: Plants (Basel). 2024 May 9;13(10):1304. doi: 10.3390/plants13101304 (PMC11124892; doi:10.3390/plants13101304)
Supplement: Supplementary file 1 [file plants-13-01304-s001.zip › plants-2927324-supplementary.pdf]

Supplementary Material

Table S1 – Details of 103 identified *CBL* genes.

Name	Gene ID	Gene location	CDS (bp)	Protein length	PI	MW (kDa)	Subcellular localization
AtCBL1	*AT1G64480*	Chr1 23948027..23949751	645	215	4.76	24.65	Mitochondrial
AtCBL2	*AT4G01420*	Chr4 580037..581518	612	204	4.56	23.52	Cytoplasmic
AtCBL3	*AT4G16350*	Chr4 9242319..9243912	681	227	5.65	26.03	Chloroplast
AtCBL4	*AT4G17615*	Chr4 9816451..9819312	642	214	4.49	24.56	Nuclear
AtCBL5	*AT4G26560*	Chr4 13406732..13407973	645	215	4.53	24.41	Nuclear
AtCBL6	*AT4G26570*	Chr4 13408421..13411070	693	231	4.55	26.54	Nuclear
AtCBL7	*AT4G33000*	Chr4 15924348..15926628	771	257	4.46	29.35	Chloroplast
AtCBL8	*AT5G24270*	Chr5 8238533..8243357	669	223	4.87	25.70	Cytoplasmic
AtCBL9	*AT5G47100*	Chr5 19129639..19132283	642	214	4.33	24.53	Nuclear
AtCBL10	*AT5G55990*	Chr5 22671451..22673855	681	227	4.66	25.81	Extracellular matrix
ZmCBL1	*Zm00001eb023890*	Chr1 96334208..96338781	642	214	4.50	24.47	Cytoplasmic
ZmCBL2	*Zm00001eb030890*	Chr1 171126747..171139985	678	226	4.55	25.79	Cytoplasmic
ZmCBL3	*Zm00001eb051150*	Chr1 260095851..260114275	762	254	4.82	29.19	Cytoplasmic
ZmCBL4	*Zm00001eb135370*	Chr3 116785120..116812850	678	226	4.60	25.92	Cytoplasmic
ZmCBL5	*Zm00001eb160330*	Chr3 226083928..226087845	963	321	5.14	35.58	Chloroplast
ZmCBL6	*Zm00001eb161280*	Chr3 228698970..228702491	657	219	4.60	25.06	Cytoplasmic
ZmCBL7	*Zm00001eb203330*	Chr4 227516915..227519211	669	223	4.54	25.47	Cytoplasmic
ZmCBL8	*Zm00001eb293130*	Chr6 170736802..170739618	672	224	4.51	25.24	Chloroplast
ZmCBL9	*Zm00001eb390900*	Chr9 119599874..119604943	642	214	4.39	24.46	Nuclear
OsCBL1	*LOC_Os01g39770*	Chr1 22427830..22430870	873	291	4.58	32.90	Chloroplast
OsCBL2	*LOC_Os01g41510*	Chr1 23498442..23502377	657	219	4.54	25.09	Mitochondrial
OsCBL3	*LOC_Os01g51420*	Chr1 29568546..29571484	801	267	4.61	29.92	Chromoplast
OsCBL4	*LOC_Os02g18880*	Chr2 11015827..11017808	642	214	4.52	24.40	Chloroplast
OsCBL5	*LOC_Os02g18930*	Chr2 11057895..11059975	642	214	4.66	24.49	Chloroplast
OsCBL6	*LOC_Os03g42840*	Chr3 23888728..23895481	678	226	4.51	25.81	Cytoplasmic

OsCBL7	*LOC_Os05g45810*	Chr5 26535877..26537911	633	211	4.72	23.92	Chloroplast
OsCBL8	*LOC_Os10g41510*	Chr10 22298889..22308687	642	214	4.40	24.50	Chloroplast
OsCBL9	*LOC_Os12g06510*	Chr12 3145027..3149567	885	295	4.79	33.48	Cytoplasmic
OsCBL10	*LOC_Os12g40510*	Chr12 25063166..25068213	678	226	4.55	25.87	Cytoplasmic
GsCBL1	*GlysoPI483463.04 G192800*	Chr4 50657900..50664524	672	224	4.44	25.26	Cytoplasmic
GsCBL2	*GlysoPI483463.05 G060000*	Chr5 6754784..6764523	642	214	4.49	24.42	Nuclear
GsCBL3	*GlysoPI483463.06 G117200*	Chr6 10809099..10816038	672	224	4.46	25.44	Cytoplasmic
GsCBL4	*GlysoPI483463.07 G009700*	Chr7 827952..830976	792	264	4.76	30.43	Cytoplasmic
GsCBL5	*GlysoPI483463.07 G226300*	Chr7 44427530..44435578	681	227	4.39	25.78	Extracellular matrix
GsCBL6	*GlysoPI483463.08 G021700*	Chr8 1919783..1922344	708	236	4.58	26.73	Nuclear
GsCBL7	*GlysoPI483463.08 G178200*	Chr8 15960299..15963649	786	262	4.64	30.12	Cytoplasmic
GsCBL8	*GlysoPI483463.08 G292900*	Chr8 45360406..45364803	681	227	4.45	26.04	Cytoplasmic
GsCBL9	*GlysoPI483463.09 G014800*	Chr9 1280304..1284347	681	227	4.64	25.93	Nuclear
GsCBL10	*GlysoPI483463.11 G035000*	Chr11 2819080..2821207	639	213	4.31	24.40	Nuclear
GsCBL11	*GlysoPI483463.17 G004000*	Chr17 1635199..1640348	681	227	4.40	25.80	Chloroplast
GsCBL12	*GlysoPI483463.17 G136100*	Chr17 12486387..12490657	819	273	4.95	31.49	Chloroplast
GsCBL13	*GlysoPI483463.17 G195000*	Chr17 5711081..5719023	777	259	4.44	29.26	Chloroplast
GsCBL14	*GlysoPI483463.18 G067300*	Chr18 7128650..7133441	681	227	7.23	26.07	Cytoplasmic
VuCBL1	*Vigun02g160400*	Chr2 30635046..30638732	639	213	4.65	24.44	Cytoplasmic
VuCBL2	*Vigun03g035200*	Chr3 2707112..2710410	663	221	4.53	25.24	Chloroplast
VuCBL3	*Vigun03g297200*	Chr3 48352954..48357092	642	214	4.57	24.48	Nuclear
VuCBL4	*Vigun03g437100*	Chr3 64073888..64080875	681	227	4.50	25.84	Chloroplast
VuCBL5	*Vigun06g066300*	Chr6 19457635..19462125	681	227	4.45	26.04	Cytoplasmic
VuCBL6	*Vigun06g148100*	Chr6 27292665..27298292	684	228	4.62	26.16	Chloroplast
VuCBL7	*Vigun09g149400*	Chr9 31299345..31304400	897	229	5.04	34.62	Nuclear
VuCBL8	*Vigun10g174100*	Chr10 39276850..39280156	777	259	4.53	29.45	Cytoplasmic
SbCBL1	*Sobic.001G294300*	Chr1 57270694..57274360	642	214	4.50	24.49	Cytoplasmic
SbCBL2	*Sobic.003G196400*	Chr3 52152819..52157258	960	320	4.93	35.87	Endoplasmic reticulum
SbCBL3	*Sobic.003G208400*	Chr3 53959654..53964864	657	219	4.68	25.11	Mitochondrial
SbCBL4	*Sobic.003G275000*	Chr3 61123590..61127013	852	284	5.14	32.29	Chloroplast
SbCBL5	*Sobic.004G130600*	Chr4 16905084..16908896	642	214	4.45	24.39	Cytoplasmic

SbCBL6	*Sobic.008G046500*	Chr8 4578265..4582802	672	224	4.68	25.68	Chloroplast
SbCBL7	*Sobic.008G152800*	Chr8 58517447..58523171	678	226	4.61	25.85	Nuclear
SbCBL8	*Sobic.009G210300*	Chr9 55662844..55665067	639	213	4.39	24.05	Chloroplast
PvCBL1	*Phvul.001G034600*	Chr1 3265192..3270335	810	270	4.51	30.13	Cytoplasmic
PvCBL2	*Phvul.002G002300*	Chr2 192377..193630	429	143	4.43	20.55	Cytoplasmic
PvCBL3	*Phvul.002G299300*	Chr2 46727451..46730153	762	242	4.62	27.65	Nuclear
PvCBL4	*Phvul.002G299400*	Chr2 46731777..46733742	645	215	4.74	24.54	Mitochondrial
PvCBL5	*Phvul.003G088500*	Chr3 17036118..17042866	681	227	4.50	25.85	Chloroplast
PvCBL6	*Phvul.003G225900*	Chr3 45718718..45722898	642	214	4.57	24.54	Mitochondrial
PvCBL7	*Phvul.006G059266*	Chr6 16722421..16726237	681	227	4.45	26.08	Cytoplasmic
PvCBL8	*Phvul.006G135900*	Chr6 24357014..24361952	684	228	4.88	26.10	Cytoplasmic
PvCBL9	*Phvul.009G052700*	Chr9 10154081..10158087	672	224	4.47	25.67	Cytoplasmic
PvCBL10	*Phvul.010G139700*	Chr10 42201713..42205147	795	265	4.63	30.16	Cytoplasmic
TaCBL1	*Traes_1AL_021B94 DEB*	Chr1A 0..4771	588	196	4.45	22.32	Cytoplasmic
TaCBL2	*Traes_1AL_4AB2B F02A*	Chr1A 2683..7530	648	216	4.57	24.67	Cytoplasmic
TaCBL3	*Traes_1AL_746797 AE4*	Chr1A 5628..8192	642	214	4.59	24.52	Mitochondrial
TaCBL4	*Traes_1BL_3BF7C 2841*	Chr1B 22236..24893	675	225	4.66	25.78	Mitochondrial
TaCBL5	*Traes_1BL_AE5EA 0313*	Chr1B 1506..3179	612	204	4.40	23.05	Mitochondrial
TaCBL6	*Traes_1BL_DA652 B828*	Chr1B 2..3639	615	205	4.41	23.28	Mitochondrial
TaCBL7	*Traes_1DL_2154C A1B7*	Chr1D 2464..5776	642	214	4.59	24.49	Chloroplast
TaCBL8	*Traes_1DL_5F63C A081*	Chr1D 1034..2868	621	207	4.41	23.55	Mitochondrial
TaCBL9	*Traes_1DL_69F530 041*	Chr1D 7..1666	249	83	4.02	9.39	Cytoplasmic
TaCBL10	*Traes_1DL_771752 3DF*	Chr1D 75..2114	582	194	4.69	22.32	Mitochondrial
TaCBL11	*Traes_1DL_E3D49 2371*	Chr1D 3173..4974	258	86	3.86	9.51	Nuclear
TaCBL12	*Traes_3AL_8456B7 FB8*	Chr3A 1840..5048	918	306	5.03	34.80	Cytoplasmic
TaCBL13	*Traes_3DL_42B337 576*	Chr3D 0..1263	246	82	4.07	9.43	Cytoplasmic
TaCBL14	*Traes_4AL_212D76 6F6*	Chr4A 3612..7099	309	103	4.91	11.92	Nuclear
TaCBL15	*Traes_4BS_EBA462 C3F*	Chr4B 3651..8333	783	261	5.80	29.51	Chloroplast
TaCBL16	*Traes_4DL_FBF95 B5AF*	Chr4D 582..4852	651	217	4.58	24.60	Cytoplasmic
TaCBL17	*Traes_4DS_C7F4F 974B*	Chr4D 2634..7773	678	226	4.57	25.73	Cytoplasmic
TaCBL18	*Traes_5AL_F2D4A CCA3*	Chr5A 408..4091	585	195	4.30	22.24	Extracellular
TaCBL19	*Traes_5BS_F12838 5EB*	Chr5B 0..5084	678	226	4.62	25.89	Cytoplasmic

Table S2 – Collinear gene pairs of *CBLs* in eight species.

Number	Gene 1	Gene 2
1	*GmCBL2*	*ZmCBL1*
2	*GmCBL2*	*ZmCBL9*
3	*GmCBL5*	*ZmCBL5*
4	*GmCBL8*	*ZmCBL5*
5	*GmCBL11*	*ZmCBL1*
6	*GmCBL11*	*ZmCBL9*
7	*GmCBL13*	*ZmCBL1*
8	*GmCBL13*	*ZmCBL9*
9	*GmCBL1*	*OsCBL4*
10	*GmCBL2*	*OsCBL8*
11	*GmCBL4*	*OsCBL4*
12	*GmCBL5*	*OsCBL3*
13	*GmCBL8*	*OsCBL3*
14	*GmCBL11*	*OsCBL8*
15	*GmCBL13*	*OsCBL8*
16	*GmCBL1*	*PvCBL3*
17	*GmCBL1*	*PvCBL9*
18	*GmCBL2*	*PvCBL2*
19	*GmCBL2*	*PvCBL6*
20	*GmCBL3*	*PvCBL3*
21	*GmCBL4*	*PvCBL3*
22	*GmCBL4*	*PvCBL9*
23	*GmCBL5*	*PvCBL1*
24	*GmCBL5*	*PvCBL10*
25	*GmCBL6*	*PvCBL5*
26	*GmCBL6*	*PvCBL8*
27	*GmCBL7*	*PvCBL3*
28	*GmCBL8*	*PvCBL10*
29	*GmCBL9*	*PvCBL7*
30	*GmCBL9*	*PvCBL8*
31	*GmCBL10*	*PvCBL5*
32	*GmCBL10*	*PvCBL7*
33	*GmCBL10*	*PvCBL8*
34	*GmCBL11*	*PvCBL2*
35	*GmCBL11*	*PvCBL6*
36	*GmCBL12*	*PvCBL5*
37	*GmCBL12*	*PvCBL8*
38	*GmCBL12*	*PvCBL7*
39	*GmCBL13*	*PvCBL2*
40	*GmCBL13*	*PvCBL6*
41	*GmCBL14*	*PvCBL1*
42	*GmCBL15*	*PvCBL8*

43	*GmCBL15*	*PvCBL7*
44	*GmCBL1*	*SbCBL5*
45	*GmCBL2*	*SbCBL1*
46	*GmCBL13*	*SbCBL1*
47	*GmCBL4*	*SbCBL5*
48	*GmCBL5*	*SbCBL4*
49	*GmCBL5*	*SbCBL2*
50	*GmCBL8*	*SbCBL4*
51	*GmCBL8*	*SbCBL2*
52	*GmCBL11*	*SbCBL1*
53	*GmCBL1*	*GsCBL1*
54	*GmCBL1*	*GsCBL3*
55	*GmCBL2*	*GsCBL2*
56	*GmCBL2*	*GsCBL10*
57	*GmCBL2*	*GsCBL12*
58	*GmCBL3*	*GsCBL1*
59	*GmCBL3*	*GsCBL3*
60	*GmCBL3*	*GsCBL6*
61	*GmCBL4*	*GsCBL1*
62	*GmCBL4*	*GsCBL3*
63	*GmCBL5*	*GsCBL4*
64	*GmCBL5*	*GsCBL7*
65	*GmCBL5*	*GsCBL13*
66	*GmCBL6*	*GsCBL5*
67	*GmCBL6*	*GsCBL9*
68	*GmCBL6*	*GsCBL11*
69	*GmCBL7*	*GsCBL1*
70	*GmCBL7*	*GsCBL3*
71	*GmCBL7*	*GsCBL6*
72	*GmCBL8*	*GsCBL4*
73	*GmCBL8*	*GsCBL7*
74	*GmCBL9*	*GsCBL8*
75	*GmCBL9*	*GsCBL9*
76	*GmCBL9*	*GsCBL14*
77	*GmCBL10*	*GsCBL5*
78	*GmCBL10*	*GsCBL8*
79	*GmCBL10*	*GsCBL9*
80	*GmCBL10*	*GsCBL11*
81	*GmCBL10*	*GsCBL14*
82	*GmCBL11*	*GsCBL2*
83	*GmCBL11*	*GsCBL10*
84	*GmCBL11*	*GsCBL12*
85	*GmCBL12*	*GsCBL5*
86	*GmCBL12*	*GsCBL8*

87	*GmCBL12*	*GsCBL9*
88	*GmCBL12*	*GsCBL11*
89	*GmCBL12*	*GsCBL14*
90	*GmCBL13*	*GsCBL2*
91	*GmCBL13*	*GsCBL10*
92	*GmCBL13*	*GsCBL12*
93	*GmCBL14*	*GsCBL13*
94	*GmCBL15*	*GsCBL8*
95	*GmCBL15*	*GsCBL9*
96	*GmCBL15*	*GsCBL14*
97	*GmCBL1*	*AtCBL8*
98	*GmCBL2*	*AtCBL9*
99	*GmCBL3*	*AtCBL1*
100	*GmCBL4*	*AtCBL8*
101	*GmCBL6*	*AtCBL3*
102	*GmCBL7*	*AtCBL1*
103	*GmCBL9*	*AtCBL5*
104	*GmCBL9*	*AtCBL10*
105	*GmCBL10*	*AtCBL3*
106	*GmCBL10*	*AtCBL10*
107	*GmCBL11*	*AtCBL9*
108	*GmCBL11*	*AtCBL4*
109	*GmCBL12*	*AtCBL3*
110	*GmCBL13*	*AtCBL9*
111	*GmCBL13*	*AtCBL4*
112	*GmCBL14*	*AtCBL7*
113	*GmCBL15*	*AtCBL5*
114	*GmCBL15*	*AtCBL10*
115	*GmCBL1*	*VuCBL2*
116	*GmCBL1*	*VuCBL7*
117	*GmCBL2*	*VuCBL1*
118	*GmCBL2*	*VuCBL3*
119	*GmCBL3*	*VuCBL2*
120	*GmCBL3*	*VuCBL7*
121	*GmCBL4*	*VuCBL2*
122	*GmCBL4*	*VuCBL7*
123	*GmCBL5*	*VuCBL8*
124	*GmCBL6*	*VuCBL4*
125	*GmCBL6*	*VuCBL5*
126	*GmCBL6*	*VuCBL6*
127	*GmCBL7*	*VuCBL2*
128	*GmCBL7*	*VuCBL7*
129	*GmCBL8*	*VuCBL8*
130	*GmCBL9*	*VuCBL4*

131	*GmCBL9*	*VuCBL5*
132	*GmCBL9*	*VuCBL6*
133	*GmCBL10*	*VuCBL4*
134	*GmCBL10*	*VuCBL5*
135	*GmCBL10*	*VuCBL6*
136	*GmCBL11*	*VuCBL1*
137	*GmCBL11*	*VuCBL3*
138	*GmCBL12*	*VuCBL4*
139	*GmCBL12*	*VuCBL6*
140	*GmCBL12*	*VuCBL5*
141	*GmCBL13*	*VuCBL1*
142	*GmCBL13*	*VuCBL3*
143	*GmCBL15*	*VuCBL4*
144	*GmCBL15*	*VuCBL6*
145	*GmCBL15*	*VuCBL5*
146	*GmCBL1*	*GmCBL4*
147	*GmCBL2*	*GmCBL11*
148	*GmCBL2*	*GmCBL13*
149	*GmCBL3*	*GmCBL7*
150	*GmCBL5*	*GmCBL8*
151	*GmCBL6*	*GmCBL10*
152	*GmCBL6*	*GmCBL12*
153	*GmCBL9*	*GmCBL10*
154	*GmCBL9*	*GmCBL15*
155	*GmCBL10*	*GmCBL12*
156	*GmCBL10*	*GmCBL15*
157	*GmCBL11*	*GmCBL12*
158	*GmCBL12*	*GmCBL15*

Table S3 – Primers used in this study.

Primer names	Sequence (5′ – 3′)	Description
GmCBL1-F	attgattagagatcttctagaATGGGCTGCTACTGCTCAACTT	Gene cloning
GmCBL1-R	gcccttgctcaccatgagctcCATCTCTGACTCTTCAATATCTGTTCTTAT	Gene cloning
GmACTIN4-qF	GTGTCAGCCATACTGTCCCCATTT	qRT-PCR
GmACTIN4-qR	GTTTCAAGCTCTTGCTCGTAATCA	qRT-PCR
GmCBL1-qF	ATGGGCTGCTACTGCTCAA	qRT-PCR
GmCBL1-qR	AATGACCCCATTGCGCTTGA	qRT-PCR
GmCBL2-qF	AAGTCAGCGAGGCAGTTTCC	qRT-PCR
GmCBL2-qR	CGTCATCGACAACAGAACTGC	qRT-PCR
GmCBL3-qF	ACCAAACAGCGAGTCGATCA	qRT-PCR
GmCBL3-qR	TAACCCCGTCGTGGATGATG	qRT-PCR
GmCBL4-qF	TCCAGCTGGCACTATTCAGG	qRT-PCR
GmCBL4-qR	CCCAAACTCAATGACCCCGT	qRT-PCR
GmCBL5-qF	GCTGTGCTCGGTGTTCATTC	qRT-PCR
GmCBL5-qR	GGCGAGGGTGAGAATGTCAT	qRT-PCR
GmCBL6-qF	TCTTGCAAGAGAGACCGTGT	qRT-PCR
GmCBL6-qR	AAGACAGAGAGAGCACGTGC	qRT-PCR
GmCBL7-qF	GCGCCACAAACACAAAAAGC	qRT-PCR
GmCBL7-qR	TCCGTCTCCTTTTGAGTCTGC	qRT-PCR
GmCBL8-qF	CTGTGCGCAGTGTTCATTCC	qRT-PCR
GmCBL8-qR	TTGAACAACTCACGCAAGGC	qRT-PCR
GmCBL9-qF	AGCACTCTACGAGCTCTTCA	qRT-PCR
GmCBL9-qR	GGAAAACGGAGAGAGCACGA	qRT-PCR
GmCBL10-qF	TGTCTTTCATCCCAGCGCAT	qRT-PCR
GmCBL10-qR	TTCAGCTAGAGTGGCAACCA	qRT-PCR
GmCBL11-qF	GAGCATCAGTAGGTCCGTCG	qRT-PCR
GmCBL11-qR	AGCGCTCTAACGAAGTCACC	qRT-PCR
GmCBL12-qF	TTGCACGAGCTCTCTCTGTC	qRT-PCR
GmCBL12-qR	TTCAGAAGGGATGGATGCCG	qRT-PCR
GmCBL13-qF	ATGGGCTGCATGAACTCGAA	qRT-PCR
GmCBL13-qR	TCCTTGCTTAGCAGTCCATCA	qRT-PCR
GmCBL14-qF	GTTCCACCATAGCCACGGAT	qRT-PCR
GmCBL14-qR	TCAAAACCTCAACCACCGCT	qRT-PCR
GmCBL15-qF	ATGTTGCGGTGCTTAGAGGG	qRT-PCR
GmCBL15-qR	TCGTCGATTACAGCACTGCT	qRT-PCR
GmCPA1-qF	GGGATTGCTTGTAGGCACCT	qRT-PCR
GmCPA1-qR	CCCAGCCGATGATGAGTACC	qRT-PCR
GmAPX1-qF	AGAAAGGGCTTCTTCGCTCC	qRT-PCR
GmAPX1-qR	GATTTTGCGAAGTCCTGGCG	qRT-PCR
GmCAT1-qF	GCGCTAGTGCAAAGGGTTTC	qRT-PCR
GmCAT1-qR	AAGGTTTCAGGGCTACCACG	qRT-PCR
GmGSH1-qF	TGAGGAACAGGCCGTACAAC	qRT-PCR
GmGSH1-qR	AAAAACCCGGTCTGGAAGCC	qRT-PCR

Primer names	Sequence (5′ – 3′)	Description
GmSOD1-qF	AGGAAGGGCTGTTGTTGTCC	qRT-PCR
GmSOD1-qR	CACAAGCTACTCTGCCACCA	qRT-PCR

Table S4 – Motif sequences identified by MEME tool.

Motif	Best possible match	Number of amino acids
Motif 1	RNGVIEFEEFVRALSVFHPNAPJEDKIEFSFRLYDLKQTGFIERZEVKZM	50
Motif 2	IIDKTFEEADTKHDGKIDKEEWKNFVLRHPSLL	33
Motif 3	EIEALYELFKKJSSSIIDDGLISKEEFQLALFKTKKKENLFADRVFDLFD	50
Motif 4	NMTLPYLKDITTTFPSFVFHS	21
Motif 5	MVQCLDGLKPLFAAIINCCDADIYKQPRGLEBPEVLARETV	41
Motif 6	ALLAESGMNLSDDVI	15
Motif 7	MGCYCSKSRKTEPDP	15
Motif 8	RLSLRSSLTFGETLCAVFIPLIGIVEALVFTFTSCFDFQSP	41
Motif 9	LASETPFTVN	10
Motif 10	HWHFNGNH	8

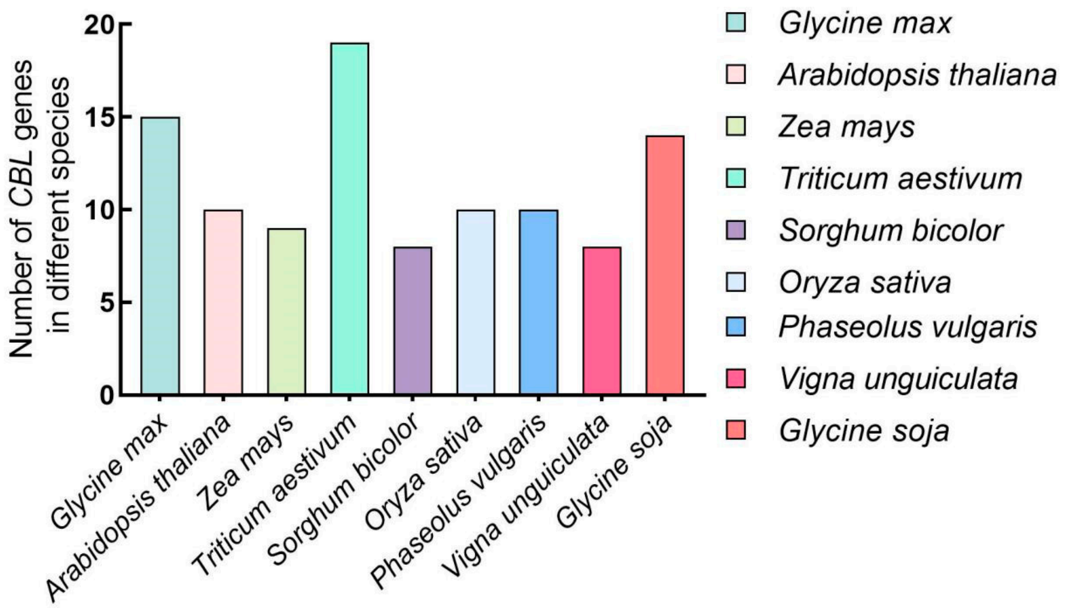

Figure. S1 – Numbers of *CBL* genes in nine plant species.

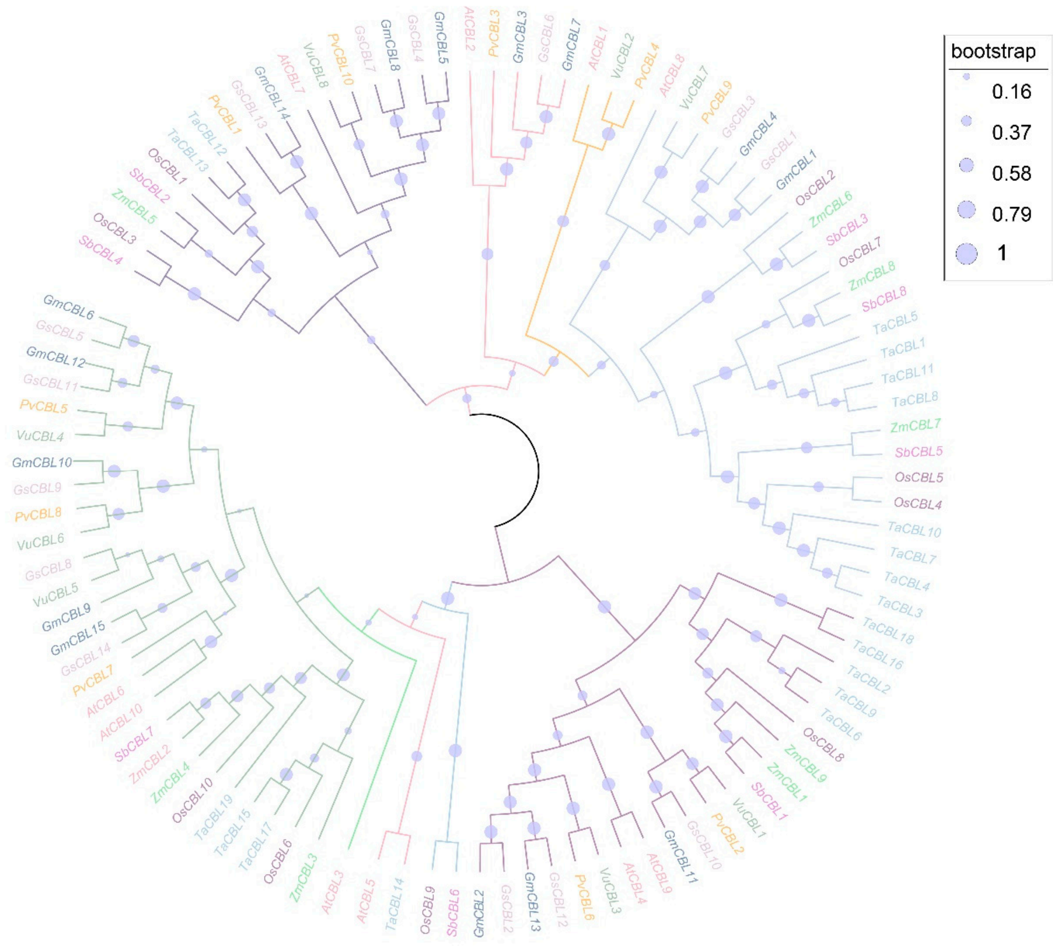

Figure. S2 – Phylogenetic tree analysis of *CBL* gene family.

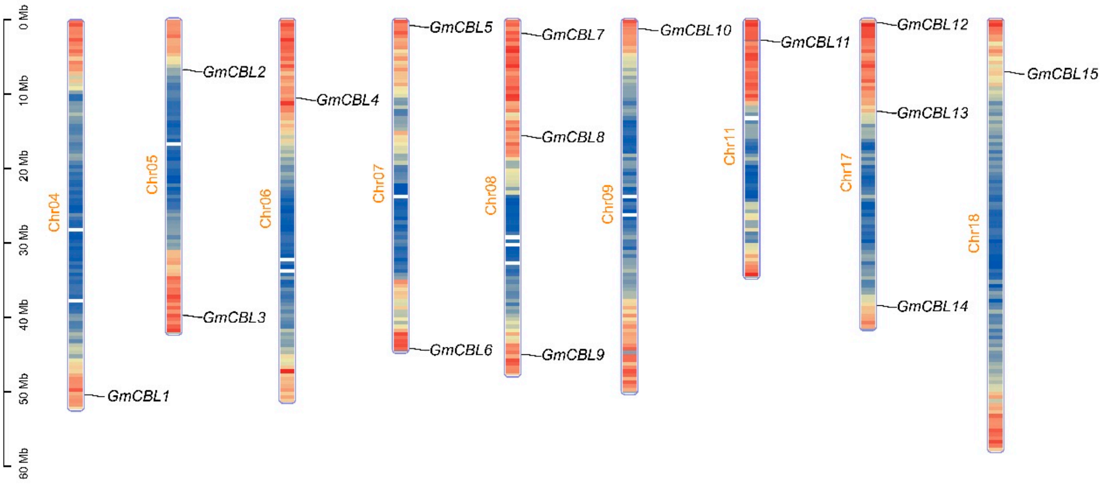

Figure. S3 – Soybean *CBL* gene chromosomal locations.

The distribution of *GmCBL* genes across soybean chromosomes is depicted, with each gene positioned according to its physical location. The left axis represents the length of each chromosome in mega base (Mb).

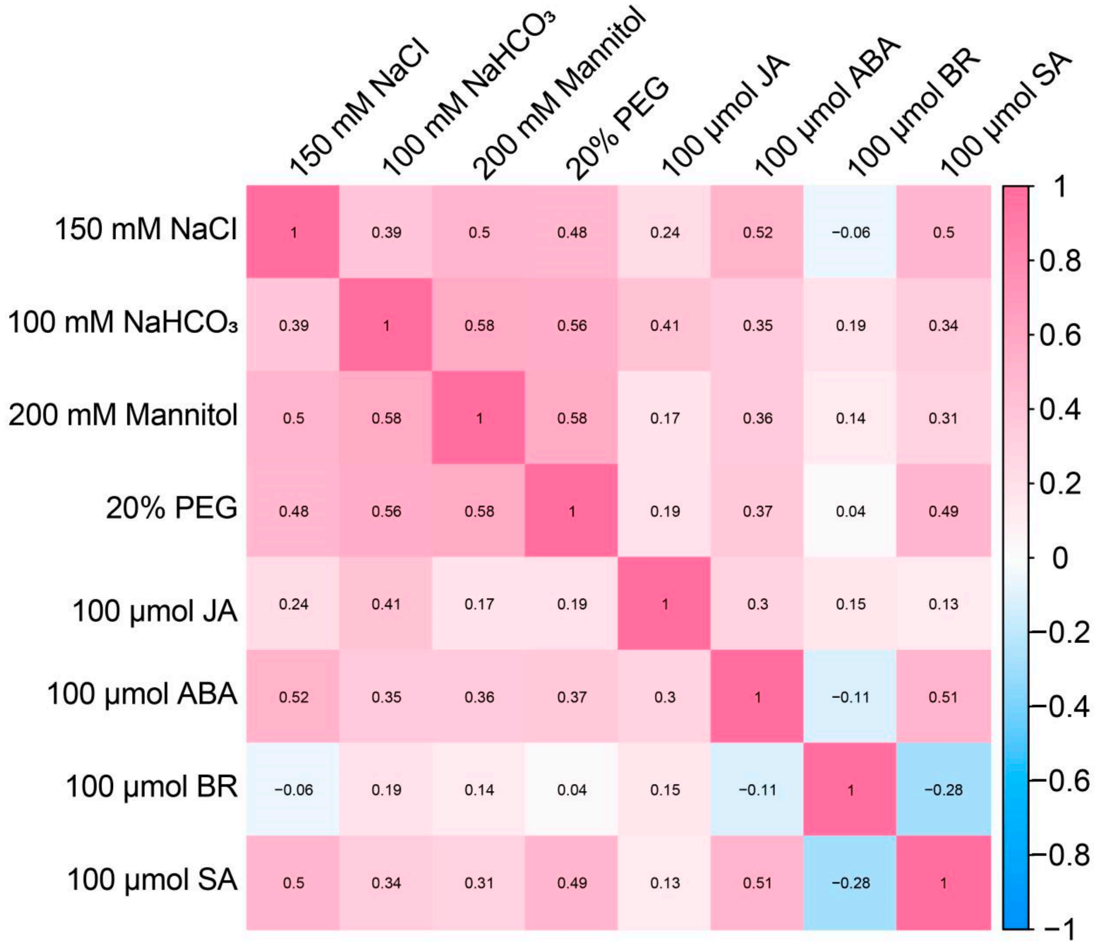

Figure. S4 – Correlation analysis of hormone stress and abiotic stress. Positive numbers represent positive correlation and negative numbers represent negative correlation. The color scale, ranging from -1.00 to 1.00 with blue representing lower values and pink representing higher values, is used to depict the normalized gene expression correlation.

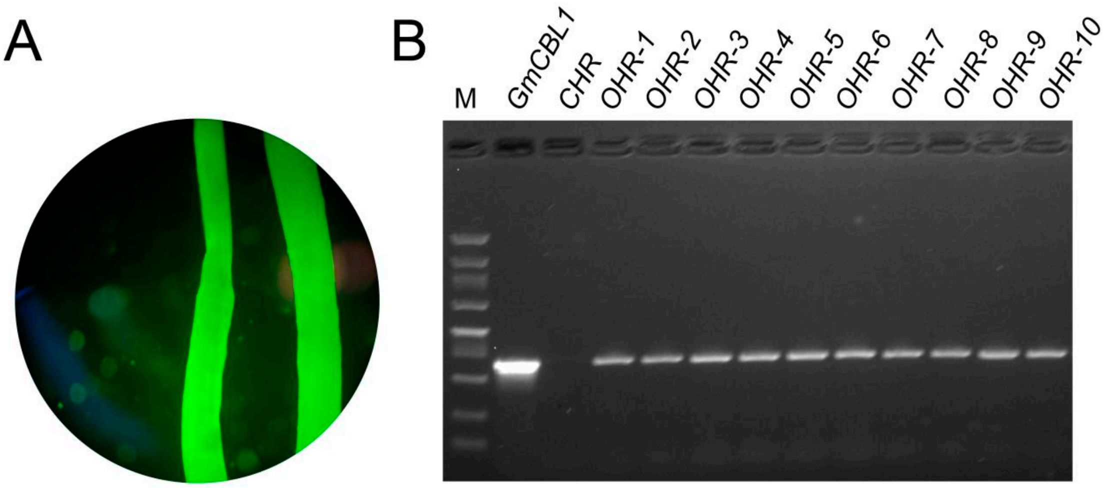

Figure. S5 – Identification of *GmCBL1* transgenic soybean hair roots.

(A) Images were captured approximately 3 weeks post-transformation to assess GFP fluorescence using a Leica stereomicroscope. (B) PCR verification of GmCBL1 in the *35s:GmCBL1*-overexpressing hairy roots (OHR1-10). M: DNA marker; Vector: the plasmid of empty vector (*pSOY I-GFP*), as the negative control; *GmCBL1*: the recombinant expression plasmid of *pSOY I-GmCBL1::GFP*, as the positive control. M: 2K PLUS.
